# Trial of a prehospital intervention with traditional Chinese medicine for acute stroke (TRACE): Protocol for a mixed-methods research study

**DOI:** 10.3389/fphar.2022.879282

**Published:** 2022-08-29

**Authors:** Yuanyuan Chen, Ziyu Tian, Shuyan Wang, Hongmei Liu, Yanfang Liu, Wei Peng, Xinxing Lai, Dahe Qi, Lingbo Kong, Ying Gao

**Affiliations:** ^1^ Dongzhimen Hospital, Beijing University of Chinese Medicine, Beijing, China; ^2^ Institute of Acupuncture and Moxibustion, China Academy of Chinese Medical Sciences, Beijing, China; ^3^ School of Life Science, Beijing University of Chinese Medicine, Beijing, China; ^4^ Beijing Emergency Medical Center, Beijing, China; ^5^ Beijing Tiantan Hospital, Capital Medical University, Beijing, China; ^6^ Beijing Hospital of Traditional Chinese Medicine, Beijing, China; ^7^ Institute for Brain Disorders, Beijing University of Chinese Medicine, Beijing, China

**Keywords:** prehospital intervention, traditional chinese medicine, xingnaojing injection, acute stroke, study protocol, mixed methods research

## Abstract

**Background:** As the only traditional Chinese medicine injection approved by the China Food and Drug Administration for use as stroke first aid in ambulances, Xingnaojing Injection (XNJI) has been widely used in cases of both acute ischemic stroke (IS) and intracerebral hemorrhage (ICH). However, there is no robust clinical evidence regarding the efficacy and safety of the early use of XNJI during stroke first aid. The main purpose of this trial is to observe whether XNJI, intravenously administered within 24 h of onset in the prehospital ambulance setting, protects against early neurological deterioration (END) on the third day of onset in patients with acute stroke.

**Methods:** The Trial of a prehospital intervention with traditional Chinese medicine for acute stroke (TRACE) is a Mixed-Methods research (MMR) study that involves a combination of quantitative and qualitative research. The quantitative research part of this project is a prospective, multicenter, observational, clinical registry study, for which we aimed to recruit 1,000 patients with acute stroke (IS and ICH). Based on our observation of whether XNJI was intravenously administered within 24 h of onset in the prehospital ambulance setting, patients with acute stroke will be divided into two groups: the exposure group comprising patients who were intravenously administered XNJI and the nonexposure group comprising patients who were not. The primary outcome is early neurological deterioration (END) on the third day of onset defined as an increase of 2 or more points in the National Institute of Health Stroke Scale score between baseline and day 3. In addition, based on the aforementioned quantitative research, qualitative research will be conducted by interviewing emergency doctors about their knowledge and attitude regarding XNJI used for stroke first aid.

**Discussion:** The results of the TRACE study will provide preliminary evidence for the relationship between XNJI used within 24 h of onset and the presence of END on the third day after stroke onset; it will aid in improving the current knowledge regarding the early use of XNJI for stroke first aid.

**Clinical Trial Registration:** clinicaltrials.gov, identifier NCT04275349

## Introduction

Stroke is the first cause of death and disability among adults in China and the second leading cause of death worldwide (Group, 2020). In 2019, among all the subtypes of stroke, ischaemic stroke (IS) had a prevalence of 62.4%, and intracerebral brain hemorrhage (ICH), of 27.9% (GBD 2019 Stroke Collaborators, 2021). Stroke, a life-threatening disease, often leads to severe central nervous system damage in case of untimely treatment (Saver, 2006; Chen et al., 2019). Early neurological deterioration (END) is a frequent and serious complication of acute stroke, with an incidence of 5%–40% (Thanvi et al., 2008; Geng et al., 2017). It is characterized by progressive deterioration of neurological function within 48-72 h after stroke (Celik et al., 2019; Kang et al., 2021). END is strongly associated with an increased risk of functional disability, mortality, and poor 3-month clinical outcomes (Kwan and Hand, 2006; Geng et al., 2017). Currently, vascular recanalization strategies, including intravenous thrombolysis (IVT) and endovascular therapy (EVT), are recommended first-line therapy options for acute ischemic stroke (AIS) (Ma et al., 2019; Powers et al., 2019). However, they only benefit a small number of patients due to narrow time windows, imaging dependence, high risk of bleeding, and limited repass rates (Wang et al., 2019). The treatment options for ICH are also limited (Schrag and Kirshner, 2020). There are no specific drugs for treating ICH, and the benefits and risks of surgical treatment are controversial (Deng and Wu, 2021; Zhang and Zhou, 2022)).

Xingnaojing injection (XNJI) is made using an extract from the An Gong Niu Huang pill, a popular and effective first-aid TCM used for acute stroke, which has been clinically applied for more than 200 years (Deng et al., 2010; Wang, 2022; Wang W. et al., 2022). Using modern pharmaceutical technology, dangerous ingredients, such as cinnabar and realgar, are removed, and four ingredients including musk, gardenia, Yujin, and borneol are retained and refined to obtain a convenient and effective water-soluble intravenous injection (Yue et al., 2019). Many studies have demonstrated the effectiveness and safety of XNJI for acute stroke (Ma et al., 2013; Shen et al., 2020; Wang et al., 2021), and the guidelines and consensus in China have recommended the use of XNJI as stroke first aid (Gao, 2016; Department of Neurology, 2018).

However, on conducting an overview of systematic reviews and meta-analyses (Tian et al., 2021), we found that none of the included studies reported on the different medication timings of XNJI use in acute stroke and on stroke severity. We also conducted a mixed-methods research (MMR) study (Tian, 2021) and found that early XNJI use within 6 h of stroke onset maybe associated with greater functional improvement. However, the appropriate initiation time of XNJI treatment is also unclear due to medical insurance restrictions and concerns regarding possible incompatibility between XNJI and IVT. A recent systematic review and meta-analysis (Wang L. et al., 2022) have also suggested that the optimum XNJI initiation time during the acute phase might be within the first 72 h after stroke onset, preferably within the first 6 h. However, due to insufficient evidence, it remains unknown whether XNJI should ideally be administered immediately after stroke onset or within 24 h. In addition, in real-world settings, the knowledge and attitude of emergency doctors regarding XNJI used for stroke first aid are also very critical. Therefore, we designed this MMR study to explore the relationship between XNJI used within 24 h of onset and the presence of END 3 days after symptom onset in patients with acute stroke.

## Methods and analysis

### Study design

**FIGURE 1:**
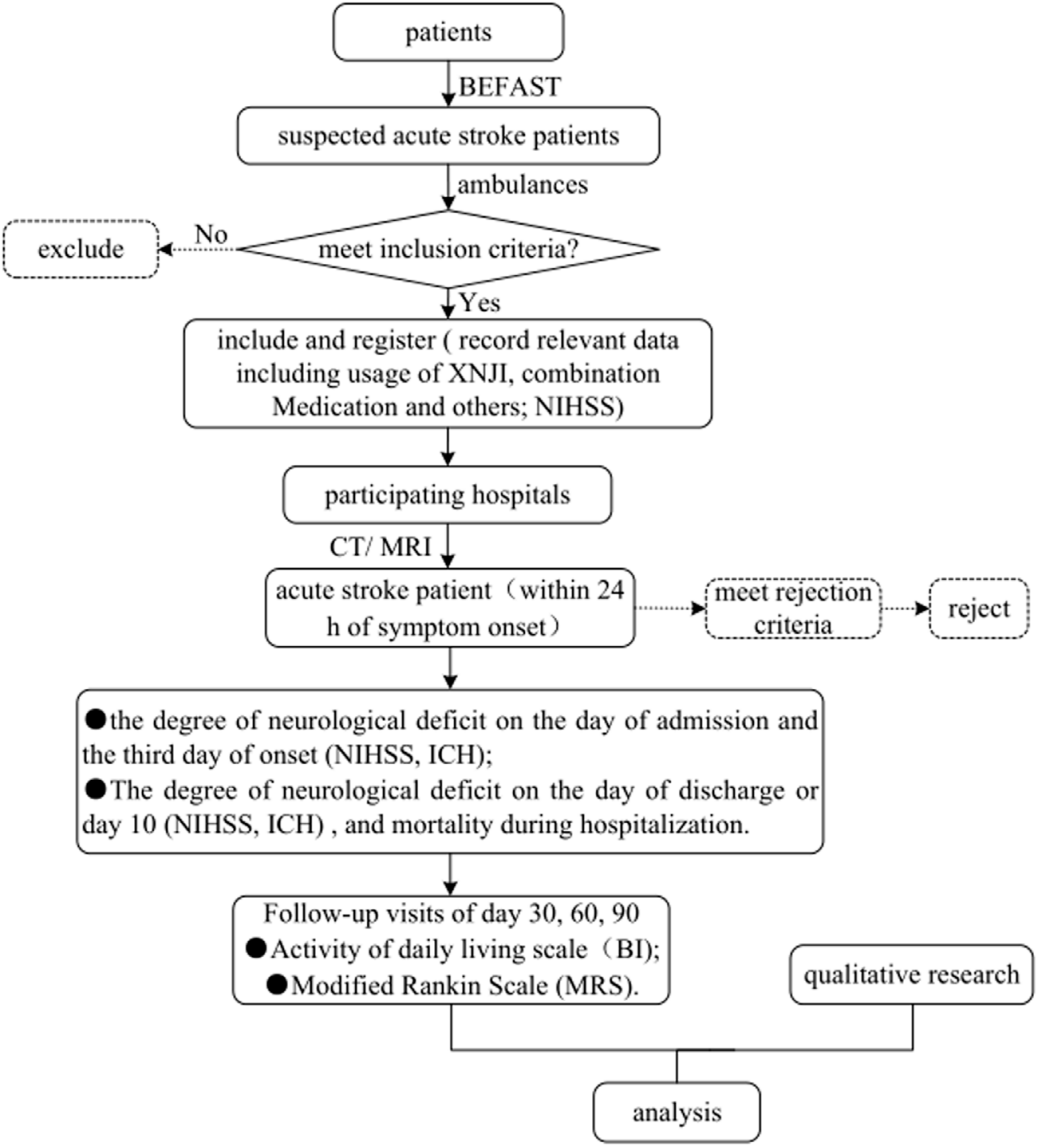
Flowchart of the TRACE study. Abbreviations: TRACE, Trial of a prehospital intervention with traditional Chinese medicine for acute stroke; BEFAST, Balance-Eyes-Face-Arms-Speech-Time; XNJI, Xingnaojing Injection; NIHSS, National Institutes of Health Stroke Scale; CT, Computed Tomography; MRI, Magnetic Resonance Imaging; ICH, intracerebral hemorrhage; BI, Barthel Index; mRS, modified Rankin Scale.

The Trial of a prehospital intervention with traditional Chinese medicine for acute stroke (TRACE) study (registered with ClinicalTrials.gov, ID: NCT04275349) will be designed as an MMR study aimed at exploring the relationship of XNJI used within 24 h of onset with the presence of END on the third day after symptom onset in patients with acute stroke. We will compare the presence of END on the third day after stroke onset between the exposure group and the nonexposure group. An overview of the study flowchart is presented in Figure 1. The registry protocol was approved by the Ethics Committee of Dongzhimen Hospital Affiliated with Beijing University of Chinese Medicine, Beijing, China (Approval number: DZMEC-KY-2019-153), and will be approved based on the requirements of the local institutional review boards of all the participating sites.

### Recruitment

Patients will be recruited consecutively from 20 participating sites, including both Western medicine hospitals and TCM hospitals in Beijing, China, which have the capability of conducting qualified research and express a proven commitment to the study. The recruitment started in March 2018 and is estimated to end in December 2022. Eligible adult patients with suspected acute stroke within 24 h of onset will be sent to the hospital by ambulance. We plan to recruit 1,000 patients with confirmed acute stroke based on the inclusion and rejection criteria. The time of stroke onset was defined as the time at which the patient was last observed to be well. Only those who meet the inclusion criteria and willingly provide written informed consent will be recruited. Since this is an observational study that involves a specific prehospital emergency intervention, it is difficult to obtain written informed consent from the patient in the prehospital ambulance setting. The patient will be asked to sign a written informed consent form in the hospital. The researchers will inform patients of the benefits and risks of participating in the research and of the need to analyze their prehospital information in addition to their in-hospital information; they will also be assured that this will not interfere with the actual clinical diagnosis and treatment. Legal guardians will sign the informed consent form on behalf of patients in a coma. If the patient or his legal guardian does not agree to the use of the diagnosis and treatment-related information in this study, the prehospital information will not be used. The participation of patients will be entirely voluntary, and their data will be strictly protected to the extent permitted by the law.

In this study, to increase the rate of identifying suspected stroke patients, the prehospital assessment scale for identifying these patients was changed from the Face-Arms-Speech-Time (FAST) scale to the Balance-Eyes-Face-Arms-Speech-Time (BEFAST) scale. Once meeting the inclusion criteria, patients will be included and registered immediately in an ambulance. However, due to the busy task and high tension in the prehospital ambulance setting, ambulance doctors are unable to exclude patients in detail. The ineligible patients will be rejected after admission. Therefore, this study has changed the exclusion criterion to the rejection criterion. 

### Inclusion criteria

•Diagnosis of suspected acute stroke
•Within 24 h of symptom onset
•Age≥18 years

### Rejection criteria

•Other diseases that lead to motor dysfunction (e.g., claudication, severe osteoarthritis, rheumatoid arthritis, and gouty arthritis)
•Other diseases or psychosis that renders outcome assessments or follow-up unlikely to be possible
•Known to be pregnant or lactating
•Non-acute stroke [result from Computed Tomography (CT) or Magnetic Resonance Imaging (MRI)].

### Intervention measures

The quantitative research part of this study will be observational. Based on our observation of whether XNJI was intravenously administered within 24 h of onset in the prehospital ambulance setting, patients with acute stroke will be divided into two groups: the exposure group comprising patients who were intravenously administered XNJI and the nonexposure group comprising patients who were not. During the study period, all relevant data will be collected at the following timepoints to facilitate a continuous dynamic observation and evaluation: during the prehospital ambulance service; within 24 h of admission; day 3 after stroke onset; day 10 after stroke onset or the day of discharge; and 30 ± 3, 60 ± 3, and 90 ± 7 days after stroke onset. This research will not interfere with the actual diagnosis and treatment of the participants. The clinicians will make decisions regarding routine treatment options based on the patient’s specific circumstances. The routine stroke treatment will follow the current national guidelines for acute Is and ICH from the Chinese Society of Neurology.

### Main variables

All variables used for outcomes evaluation of efficacy and safety are abstracted from all relevant data collected during the observation period. The main variables of this study are listed in Table 1.

**TABLE 1:** Main variables registered in the TRACE study.

Field	Variables
Visit 1 (during pre-hospital ambulance service)	
Demographics information	Name; Age; Gender; ID number
Condition, state of consciousness, and survival or death upon ambulance arrival	Time of symptom onset; Time of ambulance arrival; State of consciousness; Survival/death; Stroke assessment (BEFAST)
Usage of XNJI	Use it or not; Time of first use of XNJI; Dose
Concomitant medication	Antihypertensive drugs; Vasopressor drugs; Hypoglycemic agent; Glycemic drugs; Other drugs
Auxiliary examination	Vital signs
Indicators on health economics	Treatment costs; Inspection fees; Medicine costs; Other costs; Total costs
Prehospital evaluation	NIHSS
Visit 2 (within 24 h after admission)	
Condition, state of consciousness, and survival or death upon arrival	Time of onset, Time of arriving at the hospital; State of consciousness; Survival/death
Baseline information	Height; Weight; BMI; Being overweight or obese; Predisposing factors; Transient symptoms occurred within 2 months before stroke onset; Past medical history (strokes; CHD; atrial fibrillation; diabetes; hypertension; hyperlipidemia; apnea); Personal history (smoking; alcohol consumption); Family history; Drug allergy history; Treatment history within 2 weeks before admission
Auxiliary examination	Vital signs; Laboratory tests; Cranial CT or MRI; ECG
Complication	Lung infections; Urinary tract infections; Upper gastrointestinal bleeding; Hiccups; Dysphagia; Urine retention; Urinary incontinence; Pressure ulcers; Kidney failure; Pulmonary embolism; Epilepsy; Shoulder-hand syndrome; Post-stroke depression; Post-stroke anxiety; Others
Admission evaluation	mRS; NIHSS; ICH Scale and GCS for ICH
Visit 3 (day 3 after symptom onset)	
Auxiliary examination	Vital signs
Complications	Lung infections; Urinary tract infections; Upper gastrointestinal bleeding; Hiccups; Dysphagia; Urine retention; Urinary incontinence; Pressure ulcers; Kidney failure; Pulmonary embolism; Epilepsy; Shoulder-hand syndrome; Post-stroke depression; Post-stroke anxiety; Others
In-hospital evaluation	NIHSS; ICH Scale and GCS for ICH
Visit 4 (day 10 after symptom onset or the day of discharge)	
TOAST classification of AIS	Major artery Atherosclerotic stroke; Cardiogenic cerebral embolism; Arterial occlusive stroke or lacunar stroke; Ischemic stroke resulting from other causes; Unknown cause of ischemic stroke
Major treatment methods	IVT and EVT for AIS; Surgery (craniotomy hematoma removal; minimally invasive surgery; bone valve decompression; ventricular drainage) and others for ICH
In-hospital medication	Antihypertensive agents; Hypoglycemic agents; Lipid-lowering drugs; Anticoagulants; Hemostatic agents; Reduced cranial pressure agents; Anticoagulants agents; XNJI (start use time; stop use time; use frequency; administration duration time; dose); Other TCM (Chinese patent medicine injection agent; Chinese patent medicine oral agent; TCM soup agent)
In-hospital rehabilitation	Acupuncture and moxibustion; Massage; PT; OT; ST; Others
Auxiliary examination	Vital signs; Laboratory tests; Cranial CT or MRI; ECG
Complication	Lung infections; Urinary tract infections; Upper gastrointestinal bleeding; Hiccups; Dysphagia; Urine retention; Urinary incontinence; Pressure ulcers; Kidney failure; Pulmonary embolism; Epilepsy; Shoulder-hand syndrome; Post-stroke depression; Post-stroke anxiety; Others
Indicators on health economics	Hospitalization costs; Treatment costs; Inspection fees; Medicine costs; Other costs; Total costs; Number of days of hospitalization
Discharge diagnosis	
In-hospital mortality	
Discharge evaluation	NIHSS; ICH Scale and GCS for ICH
Visit 5 (day 30 ± 3 after symptom onset)	
Follow-up evaluation	mRS; BI
Visit 6 (day 60 ± 3 after symptom onset)	
Follow-up evaluation	mRS; BI
Visit 7 (day 90 ± 7 after symptom onset)	
Basic medication	Antithrombotics agents; Anticoagulants agents; Lipid-lowering drugs; Antihypertensive agents; Hypoglycemic agents; TCM
Rehabilitation	Acupuncture and moxibustion; Rehabilitation exercise
Follow-up evaluation	mRS; BI
AEs (during the observation period)	Start time; Symptoms/signs; Intensity; Attack frequency; Duration and termination time; Laboratory indicators; Treatment methods and outcomes; Follow-up results

Note: Field and variables not specifically specified will be applied to all acute stroke patients (IS and ICH). Abbreviations: TRACE, The Trial of a prehospital intervention with traditional Chinese medicine for acute stroke; ID, identity card; BEFAST, Balance-Eyes-Face-Arms-Speech-Time; XNJI, Xingnaojing Injection; NIHSS, National Institutes of Health Stroke Scale; BMI, Body Mass Index; CHD, Coronary Heart Disease; CT, Computed Tomography; MRI, Magnetic Resonance Imaging; ECG, electrocardiogram; mRS, modified Rankin Scale; ICH, intracerebral hemorrhage; GCS, Glasgow Coma Scale; TOAST, Trial of Org 10172 in Acute Stroke Treatment; IVT, intravenous thrombolytic therapy; EVT, endovascular treatment; TCM, traditional Chinese medicine; PT, Physical therapy; OT, Occupational therapy; ST, Speech training; BI, Barthel Index; AEs, Adverse Events.

### Data collection and management

Through a prospectively designed electronic case registration form (eCRF), trained ambulance doctors will collect the prehospital diagnosis- and treatment-related information of the patient and will perform assessments using the National Institute of Health Stroke Scale (NIHSS) score through a face-to-face interview within 24 h after stroke onset during the prehospital ambulance service. Trained researchers appointed at each participating site will collect in-hospital data as follows: conditions state of consciousness, and survival or death on the day of admission; baseline information on the day of admission; the Trial of Org 10172 in Acute Stroke Treatment (TOAST) classification of AIS; major treatment methods; in-hospital medication; rehabilitation; auxiliary examination; complication; in-hospital mortality; discharge diagnosis and Indicators on health economics; they will also perform assessments using the NIHSS scale, ICH scale, Glasgow Coma Scale (GCS), and mRS during a face-to-face interview during hospitalization.

In this study, the data entry process will be refined, and different responsibilities and authorities of prehospital and in-hospital personnel will be emphasized. In the prehospital ambulance setting, the researcher will need to record the prehospital ambulance data of the patient promptly and fill in, complete, and submit the prehospital module before arriving at the hospital to hand over to the patient. Once the patient is admitted to the hospital, the information collected in the prehospital ambulance setting will be submitted by default and will not be modified again. After admission, the researcher in the receiving hospital will record the diagnosis and treatment data in the hospital timely, accurately, completely, and clearly in the eCRF according to the original observation record. Case registration must be completed for each selected patient; data collection and management will be conducted using an electronic data capture (EDC) system (http://1-dao.net:13579); the researchers and research assistants are responsible for data entry and submission by logging in the mobile terminal information platform. If the clinical supervisor has questions regarding the data recorded in the eCRF, the research assistants will consult with the researchers, who will answer and return as soon as possible, and then modify, confirm, and input the data. The research assistant will assist in completing the q & A work and fill out the data entry Q & A form. If the data collected after admission are found to be incorrect, modifications can be made with the help of a form comprising online questions and answers; the supervisor will thoroughly check all the data, raises questions, and coordinate with the researchers to resolve any concerns. The main goal of the verification process is to confirm whether data collection via the eCRF is timely, complete, objective, and accurate. The consistency of data and source files (photo of uploaded laboratory examination/function check report) will be checked, and a clinical explanation will be sought in case of any laboratory abnormalities. The supervisor will also check whether modifications of data collected through the eCRF comply with the specifications and note the reason for the modification; further, the collected data will be reconfirmed to ensure no logic errors. The electronic data entry system is set up with the necessary legal values and logical verification rules, whereby it alerts the researcher in case of possible outliers or data logic errors. The supervisor will regularly send data-related questions to the researchers in the participating centers and will follow up regarding the completion of the responses. After completing the data entry, the researcher will upload the data to the data center. The study data will be downloaded from the data center and transferred to the research team leader, sponsor, and statistical analyst. Written records of all the data handover processes will be maintained. The responsible unit and statistical analyst will lock the data, and no further revisions will be performed. Problems found after data locking will be corrected in the statistical analysis program after confirmation. Finally, after all the research data are entered and locked, the database will be submitted to the statistical analysts, who will perform statistical analyses according to the requirements outlined in the statistical plan.

### Follow-up procedures

Independent interviewers with a background in neurology will be trained in a standard operating procedure (SOP) for contacting patients. The interviewers will regularly follow up with the patients over the telephone using standard interview forms in the eCRF at 30 ± 3, 60 ± 3, and 90 ± 7 days after stroke onset. The standard interview forms in the eCRF include the mRS, BI, basic medication-related questions, and rehabilitation-related questions. All interviews will be recorded and saved. Patients will have the right to withdraw from the trial at any time without reason, punishment, or loss of interest, and their medical treatments will not be affected. Participants who do not formally withdraw from the trial but no longer attend follow-up sessions will also be regarded as having withdrawn from the trial. The data of these participants will be considered valid and will not be replaced. While respecting the individual rights of the participants, researchers should try their best to understand the reasons for withdrawal to better protect the participants. The participants who drop out due to AEs should be followed up until their physical status is stable, while the other participants who dropped out should not be followed up.

### Types of outcomes

#### Efficacy outcomes

The primary outcome is END on the third day of onset defined as an increase of 2 or more points in the NIHISS score between baseline and day 3. The secondary outcomes include the rates of stroke-related and any-cause deaths during hospitalization, differences in the changes in the neurological deficit degree, risk grade of ICH evaluated through changes in the ICH scale score from baseline to day 10 or the day of discharge, the proportion of patients with activities of daily living with a Barthel Index score of ≥90, and the proportion of independent patients defined by a modified Rankin Scale score ≤2 at 30 ± 3, 60 ± 3, and 90 ± 7 days after stroke onset. A complete list of the efficacy outcomes analyzed in the TRACE study is presented in Table 2.

**TABLE 2:** | The complete list of efficacy outcomes analyzed in the TRACE study.


Primary outcome
1. Early neurological deterioration# change
Early neurologic deterioration (END) is defined as an increase of 2 points or more in the National Institute of Health Stroke Scale (NIHSS) score between baseline and 3 days. The NIHSS score ranges from 0 (best score) to 42 (worst score). [Time Frame: between baseline and 3 days]
Secondary outcomes
1. Rate of stroke-related deaths and deaths from any cause Rate of stroke-related deaths and deaths from any cause during hospitalization [Time Frame: during hospitalization] 2. Neurological deficit Comparison of the change of degree of neurological deficit evaluated by the NIHSS [Time Frame: baseline and day 10 or the day of discharge] 4. Risk grade of a cerebral hemorrhage Comparison of the change of risk grade of Intracranial hemorrhage evaluated by Intracerebral Haemorrhage (ICH) scale. The ICH score ranges from 0 (best score) to 6 (worst score). [Time Frame: baseline and day 10 or the day of discharge] 5. Activities of daily living Activities of daily living will be measured by Barthel Index (BI) score. The proportion of patients with a BI score of ≥ 90. The BI score ranges from 0 (worst score) to 100 (best score). [Time Frame: 30, 60, and 90 days] 6. The proportion of patients’ independent The proportion of patients’ independence is defined as a modified Rankin Scale (mRS) score of ≤ 2. The mRS score ranges from 0 (best score) to 6 (worst score). [Time Frame: 30, 60, and 90 days]

Note: Baseline score refers to the NIHISS and ICH scores within 24 h of symptom onset (assessed in a prehospital ambulance or after admission). Abbreviations: TRACE, Trial of a prehospital intervention with traditional Chinese medicine for acute stroke; NIHSS, National Institutes of Health Stroke Scale; ICH, intracerebral hemorrhage; BI, Barthel Index; mRS, modified Rankin Scale.

#### Safety outcomes

We defined an AE as any unfavorable and unintended sign, symptom, or disease that develops in the participants in both groups during the trial period regardless of whether or not it is related to the XNJI treatment (Lai et al., 2017). Safety outcomes will be assessed by routine physical and laboratory examinations and AEs recording during the observation period. Because the methodology of the study does not interfere with the actual clinical diagnosis and treatment, we changed our approach from AEs processing to AEs recording. If AEs occur during hospitalization, it will not need to be reported; however, relevant information (start time; symptoms/signs; intensity; attack frequency, duration, and termination time; laboratory indicators; treatment methods and outcomes; follow-up results; etc.) will have to be accurately recorded. The use of all concomitant medications should be recorded in detail. If any treatment is required, a record should be made regarding the treatment administered.

### Quality control and monitoring

Before registration, several approaches will be implemented to ensure coordination among the different centers to avoid the potential heterogeneity of data. First, all the research personnel at each center and independent interviewers will be trained so that they understand the clinical trial protocol and comply with the SOP for consecutive patient screening and recruitment, obtaining informed consent, and collecting data (scale filling, scale evaluation, eCRF filling), etc; this will be achieved through centralized on-site training, videoconferences, and training videos. Second, special quality auditors will be set up in each subcenter and will conduct regular in-institution quality audits, which will include the subject registration process, the implementation of registration standards, data authenticity and traceability, data collection integrity, etc. Third, an independent and professional third-party supervision agency will perform the main supervision task of the research group. The monitoring personnel will set up the monitoring task, frequency, and time points according to the research risks; they will regularly analyze and deal with the research risks under the guidance or approval of the group leadership unit. The data monitoring process is divided into two parts: on-site monitoring and remote network center monitoring. Remote monitoring will be carried out based on the project management platform and EDC system. In this study, the data quality monitoring will mainly be conducted remotely, and standardization, compliance, and data authenticity monitoring during the research process will mainly be conducted on-site. The supervisor will contact the research assistants, researchers, and central quality control personnel from time to time to ensure the implementation of the research and quality control process. A clinical research associate (equivalent to a clinical study monitor, CRA) will visit the research centers regularly or irregularly for site inspection. The CRA will be responsible for tasks such as the following: to check whether the documents recorded are timely, complete, accurate, and true; to check whether the signing of the informed consent form complies with good clinical practice (GCP) and relevant regulatory requirements; to ensure that all the data collected via the eCRF are consistent with the original data, such as those in the hospitalization medical records; and to ensure that all AEs are recorded.

### Sample size calculation

According to the definition of the dominant population, at least one particular subgroup will have a positive outcome in terms of the primary efficacy index. A previous study conducted in Germany reported that approximately 13% of patients exhibited END on the third day after stroke onset (an increase of 2 or more points in the NIHSS score). Data obtained from our previous research as well as other domestic research showed that the rate of END on the third day after stroke onset was approximately 6–8%. The sample size was calculated on the following assumptions: the END rate in patients who did not receive the TCM intervention was 13% based on data from studies conducted overseas, and the END rate in patients who received the TCM intervention was 7% based on domestic data. Since there is no formula meant specifically for calculating the sample size in registry studies, we decided to use the one-sided difference test based on the statistical analysis method and formula for calculating the sample size in cohort studies. Consequently, the following formula, where α = 0.05 and β = 0.2, was used:

$$\text{n}={\left[\frac{Z_{1}-\alpha \sqrt{2\text{pq}}+Z_{1}-\beta \sqrt{{\text{p}}_{1}\left(1-{\text{p}}_{1}\right)+{\text{p}}_{2}\left(1-{\text{p}}_{2}\right)}}{{\text{p}}_{1}{-\text{p}}_{2}}\right]}^{2}.$$

The smallest sample size for a single cohort was estimated to be 426 patients; therefore, a minimum of 852 patients must be included. Considering the uneven distribution of exposure factors among patients and the inability of some patients to attend all the visits through the 90 days, the sample size was increased to 1,000 cases, which is an increase of 17% on the original base size.

### Qualitative research

Qualitative research will be conducted based on the results of the quantitative research. The emergency doctors participating in this study will be interviewed via one-to-one semi-structured interviews or group interviews, lasting approximately 60 min/person. The number of interviewers will be determined based on the results of three to five pre-interviews. According to the degree of information saturation, approximately 20 doctors will be interviewed. The interview will be mainly about the knowledge and attitude of the emergency doctors regarding XNJI used for stroke first aid. The process is as follows:

• Interview sampling: Purposive sampling. Depending on the degree of information saturation, approximately 20 individuals will be interviewed based on the results of the pre-interviews.
• Interview place: Beijing 120 Emergency Center.
• Interview time: Approximately 60 min/person.
• Data collection: A basic personal questionnaire will be used to collect data, such as the name, sex, age, education background, professional title, years of first aid work, professional physician certificate category, etc. During this research process, the content of the interviews of participating doctors will be documented in detail to help researchers in understanding the respondent’s basic information.

### Data processing and analysis

The mean (Mean), standard mean difference (SD), median (Median), interquartile ranges, minimum (Min), and maximum (Max) will be used to summarize continuous data, while rate or percentage will be used for categorical data. Between-group comparisons will be conducted using parametric or non-parametric tests, as appropriate. Pearson’s chi-squared test is used for the count data, and the results are expressed as rate or percentage (%). Measured data, if data are normally distributed and the variance is homogeneous, the parametric test (*t*-test) will be used and results will be expressed as mean ± SD; If the data are not normally distributed and still do not meet the requirements for the parametric test after data conversion, the non-parametric test (Wilcoxon rank sum test or Mann-Whitney U test) will be used and results will be expressed as median (Median).

As the primary analysis, a comparison of the presence of END on the third day after stroke onset will be conducted between the two groups of patients—the exposure group and the nonexposure group—using Pearson’s chi-squared test. The secondary outcome analyses will involve the rate of stroke-related and any-cause deaths, differences in the NIHISS scores, differences in the ICH scale scores, proportion of patients with BI scores ≥90, and proportion of patients with mRS scores ≤2. These analyses will be performed according to the standard statistical principles for comparing parametric or non-parametric distributions, as appropriate. During the analysis, multivariate linear regression analysis is performed for the influencing factors that are statistically significant in the univariate analysis. The full analysis set will be used in the efficacy correlation analysis in this study. The safety analysis set will be used in the safety analysis. The full analysis set refers to the set of eligible cases and detached cases but not eliminated cases. The safety analysis set will consist of safety indicators for all enrolled subjects with post-medication safety evaluation data; the number of subjects with missing data will be reported. All statistical tests will be one-sided, and statistical significance will be set at *p* < 0.05. SAS software, version 9.2 (SAS Institute, Inc., Cary, NC, United States), will be used to perform the statistical analyses.

## Discussion

A wealth of active clinical research is being conducted in stroke medicine driven by the significant public health and socioeconomic implications of this common disease (Hurford et al., 2020). Given that current guideline-recommended treatments for AIS and ICH are limited, there remains a need for a more effective and safe prehospital treatment, which could reduce the impact of treatment delay on efficacy. At the same time, to close the gap in stroke care between countries, we should emphasize the importance of expanding evidence-based stroke first aid strategies that are culturally and environmentally appropriate. For thousands of years, traditional Chinese medicine (TCM) has been widely used as an important and effective treatment for stroke in China, but the evidence supporting its use is insufficient (Wu et al., 2007; Wu et al., 2019). Many previous studies have demonstrated the efficacy and safety of XNJI in acute stroke. However, due to the low level of evidence, the optimum timing for initiating XNJI treatment during stroke first aid is still unclear. Therefore, we designed this MMR study to explore the relationship between XNJI used within 24 h after stroke onset and the presence of END on the third day after symptom onset in patients with acute stroke. Evaluating the efficacy and safety of complex TCM interventions involves multiple associations (Feng et al., 2021). Fortunately, the MMR design is suitable for an individualized evaluation of TCM in the actual clinical setting and enables us to explore and interpret research-related concerns from multiple dimensions in the real-world setting (Qiu et al., 2020). A potential advantage of the MMR design is that the effect and safety of XNJI in our trial will resemble those in clinical practice.

However, there are several limitations to this study. First, it will remain unknown whether XNJI can improve prognosis and reduce mortality in patients with acute stroke over longer periods due to the relatively short follow-up period of 3 months. Second, the eCRF that will be used in this study is a pre-designed module, which may lead to the loss of some key information; this form will need to be improved in future studies. Furthermore, because this study will be performed in Beijing China, it is uncertain whether the clinical efficacy of XNJI would be similar in other regions in China and other countries. Therefore, future randomized controlled trials with more rigorous study design, robust quality control, longer follow-up periods, larger cohort sizes, and multicenter or international collaboration are still needed to obtain additional high-quality evidence supporting the use of XNJI as stroke first aid.

To the best of our knowledge, the TRACE study will provide preliminary evidence for the relationship between XNJI used within 24 h of onset and the presence of END on the third day after stroke onset in real-world settings; consequently, it will aid in improving the current knowledge regarding the early use of XNJI for stroke first aid.

## Abbreviations

AEs, adverse events; AIS, acute ischemic stroke; BEFAST, Balance-Eyes-Face-Arms-Speech-Time; BI, Barthel Index; EDC, electronic data capture; END, early neurological deterioration; EVT, endovascular therapy; GCS, Glasgow Coma Scale; IS, ischemic stroke; ICH, intracerebral brain hemorrhage; IVT, intravenous thrombolysis; MMR, mixed-methods research; eCRF, electronic case registration form; NIHSS, National Institute of Health Stroke Scale; ECG, electrocardiographic; CRA, clinical research associate; mRS, modified Rankin Scale; SOP, standard operating procedure; TIFs, telephone interview forms; TCM, traditional Chinese medicine; TRACE, Trial of a prehospital intervention with traditional Chinese medicine for acute stroke; TOAST, Trial of Org 10172 in Acute Stroke Treatment; XNJI, Xingnaojing Injection.
